# Selection of SNP subsets for association studies in candidate genes: comparison of the power of different strategies to detect single disease susceptibility locus effects

**DOI:** 10.1186/1471-2156-7-20

**Published:** 2006-04-05

**Authors:** Emmanuelle Cousin, Jean-Francois Deleuze, Emmanuelle Genin

**Affiliations:** 1 Sanofi-Aventis, Evry Genetics Centre, 2 rue Gaston Crémieux CP5705, 91057 Evry, France; 2 INSERM U 535, Hôpital Paul Brousse, secteur Jaune, BP 1000, 94817 Villejuif, France

## Abstract

**Background:**

The recent advances in genotyping and molecular techniques have greatly increased the knowledge of the human genome structure. Millions of polymorphisms are reported and freely available in public databases. As a result, there is now a need to identify among all these data, the relevant markers for genetic association studies. Recently, several methods have been published to select subsets of markers, usually Single Nucleotide Polymorphisms (SNPs), that best represent genetic polymorphisms in the studied candidate gene or region.

**Results:**

In this paper, we compared four of these selection methods, two based on haplotype information and two based on pairwise linkage disequilibrium (LD). The methods were applied to the genotype data on twenty genes with different patterns of LD and different numbers of SNPs. A measure of the efficiency of the different methods to select SNPs was obtained by comparing, for each gene and under several single disease susceptibility models, the power to detect an association that will be achieved with the selected SNP subsets.

**Conclusion:**

None of the four selection methods stands out systematically from the others. Methods based on pairwise LD information turn out to be the most interesting methods in a context of association study in candidate gene. In a context where the number of SNPs to be tested in a given region needs to be more limited, as in large-scale studies or wide genome scans, one of the two methods based on haplotype information, would be more suitable.

## Background

The high density of Single-Nucleotide Polymorphisms (SNPs) throughout the genome and the easiness of their genotyping have made these markers a widely used tool for association studies in candidate genes. During the last few years, new developments in genetics have enhanced even more their privileged situation. Large-scale investments, like the Human Genome Project and the HapMap project [1-3], have provided new information about gene function and improved the knowledge on the human genome variability. Hundreds of thousands of SNPs are now reported in public or private databases [2-4] and the number of markers described within a candidate gene often reaches several tens. Moreover, with the development of high-throughput genotyping platforms, the cost of genotyping is no longer as expensive and restrictive as it was a few years ago. However, typing all the SNPs identified within a candidate gene implies a large consumption of DNA and multiple-testing problems. Finding ways to optimize the use of markers in association tests has become an important research topic.

In this context, different decision rules have been proposed to select, among the set of SNPs identified within a candidate region, subsets of markers to genotype and use in association testing. Selection methods can roughly be divided into two categories, depending on whether they rely on the haplotype distribution or on the pairwise linkage disequilibrium. The first methods define the best subset of SNPs within a gene as the set of markers that best predicts the haplotype diversity. The second methods are based on pairwise linkage disequilibrium (LD) and select the markers that best represent the different LD groups within the gene.

In this paper, we present a comparative study of four selection methods: two haplotype-based methods: the htSNP method [5] and the tagSNP method [6], and two LD based methods [7,8]. In the following, these four methods will be referred to as Method I, II, III and IV. These methods were applied to the genotype data on twenty candidate genes, all available at the University of Washington-Fred Hutchinson Cancer Research Center Web site [9]. The SNP subsets proposed by the different methods were compared by estimating their power to detect an association under different genetic models and disease susceptibility (DS) site assumptions.

## Results

The four selection methods were applied to the genotype data on twenty candidate genes with various numbers of SNPs and LD patterns (the main characteristics of these candidate genes are presented in Additional file 1). For each gene, the number of SNPs selected by the different methods is given in Table 1. For two genes, FGL2 and PROCR, the set of markers selected was exactly the same with the four methods. Otherwise, most of the selected subsets are different and for a same gene, the number of SNPs selected may greatly vary. For example, depending on the method, 8 to 21 SNPs are selected in C3AR1 and 2 to 6 in VTN. For Method IV (Carlson et al.), as explained in the Methods section, two different thresholds for the selection criteria r^2 ^were considered: 0.5 and 0.8. As expected, the higher the r^2 ^threshold, the more the number of SNPs selected.

**Table 1:** Number of SNPs selected by the different methods

	Number of SNPs selected				
	Method I (Johnson et al.)	Method II (Stram et al.)	Method III (Cousin et al.)	Method IV 0.5 (Carlson et al.)	Method IV 0.8 (Carlson et al.)
C3AR1 – 21 SNPs -	8	21	10	11	11
CCR2 – 22 SNPs -	5	8	10	9	10
CEBPB – 10 SNPs -	5	5	4	5	6
CSF2 – 17 SNPs -	6	9	9	8	9
FCN3 – 14 SNPs -	8	10	10	10	10
FGL2 – 6 SNPs -	4	4	4	4	4
IFNG – 13 SNPs -	8	13	9	7	9
IL13 – 16 SNPs -	6	11	11	10	11
IL24 – 24 SNPs -	7	11	8	11	11
IL9 – 14 SNPs -	6	7	6	6	7
LTA – 19 SNPs -	8	11	9	8	12
LTB – 7 SNPs -	4	6	6	6	6
MC1R – 22 SNPs -	7	10	6	9	14
PLAU – 23 SNPs -	6	9	4	8	10
PROCR – 13 SNPs -	6	6	6	6	6
RELA – 12 SNPs -	5	6	7	7	7
SERPINC1 – 27 SNPs -	6	13	6	7	9
TNF – 12 SNPs -	4	5	3	4	6
TRADD – 11 SNPs -	5	8	8	7	7
VTN – 15 SNPs -	5	6	2	5	6

In Table 2, a ranking of the different methods based on the number of SNPs selected is proposed (rank 1 for the method selecting the smallest number of SNPs, and rank 5 for the one selecting the largest number of SNPs). Method I (Johnson et al.) is found to select smaller SNP subsets than the other methods. Surprisingly, although it is based on haplotype information as Method I, Method II (Stram et al.) is in fact much closer to Method IV (threshold at 0.8) in terms of number of SNPs selected. These two methods usually select more SNPs than Method III and Method IV (with a threshold at 0.5) that select subsets of very similar sizes.

**Table 2:** Classification of the selection methods for the number of SNPs selected

	Rank for number of SNPs selected				
	Method I (Johnson et al.)	Method II (Stram et al.)	Method III (Cousin et al.)	Method IV 0.5 (Carlson et al.)	Method IV 0.8 (Carlson et al.)
C3AR1	1	5	2	3	3
CCR2	1	2	4	3	4
CEBPB	2	2	1	2	5
CSF2	1	3	3	2	3
FCN3	1	2	2	2	2
FGL2	1	1	1	1	1
IFNG	2	5	3	1	3
IL13	1	3	3	2	3
IL24	1	3	2	3	3
IL9	1	4	1	1	4
LTA	1	4	3	1	5
LTB	1	2	2	2	2
MC1R	2	4	1	3	5
PLAU	2	4	1	3	5
PROCR	1	1	1	1	1
RELA	1	2	3	3	3
SERPINC1	1	5	1	3	4
TNF	2	4	1	2	5
TRADD	1	4	4	2	2
VTN	2	4	1	2	4

total	26	64	40	42	67
final rank	1	4	2	3	5

For each gene, we estimated the power of the selected subsets to detect an association. We considered both single locus tests and haplotypic tests. As detailed in the Methods section, the power of a given subset was estimated by taking the average over a range of 55 predefined genetic models and over the different possible DS loci within the gene. For Method IV, only the most powerful combination among the different proposed ones was considered. Indeed for a given gene, this method usually proposes several combinations of SNPs that often differ for SNPs in complete LD so that in fact they lead to very similar powers.

The detailed results of the power computations are available in Additional file 2. Rather than the power values, we chose to report in this article the rank of the selection methods for each gene, with rank 1 for the method selecting the subset with the highest power of detection (Table 3). For both single locus and haplotypic tests, subsets selected by the two methods based on haplotypes (Method I and II) are less powerful to detect an association. On the contrary, subsets selected by the methods based on pairwise information are very similar to each other in terms of power and more powerful. For most of the genes, the subsets selected by these methods are nested into each other and differ only in one or two SNPs, explaining the similar power results. Interestingly, for both haplotypic and single locus tests, the best results are obtained with Method IV but with different thresholds. Indeed, Method IV with a 0.5 threshold is ranked first for haplotypic tests and third for single locus tests whereas Method IV with a 0.8 threshold is ranked first for single locus tests and second for haplotypic tests. If we look jointly at Tables 2 and 3, we can see that, the extra(s) SNP(s) selected by Method IV with a 0.8 threshold or by Method III (Cousin et al.) seem to decrease the power of haplotypic tests, see for example IFNG and IL13 but increase the power of single locus tests. For Method I (Johnson et al.), low power values are observed due to a very drastic selection that results in a loss of information. Within VTN for example, Method I only selects a subset of 2 SNPs that is clearly less powerful for both single locus and haplotypic tests than the 5 SNP subset selected by Method IV (with a 0.5 threshold). A similar trend is observed in some of the studied genes with Method III and in particular genes with SNPs in high LD, see for example TNF or VTN. The bad performance of Method II (Stram et al.) in terms of power is not due to the selection of too small subsets but rather to the selection of subsets of SNPs that are not representative enough of the overall SNPs present in the gene. This is well illustrated for CSF2 and IL9 where the method selects the same number of SNPs as Method IV (with a 0.8 threshold) but is significantly less powerful.

**Table 3:** Classification of the selection methods for the power of the subsets to detect an association

	Method I (Johnson et al.)		Method II (Stram et al.)		Method III (Cousin et al.)		Method IV 0.5 (Carlson et al.)		Method IV 0.8 (Carlson et al.)	
					
	haplot. tests^a^	SL tests^b^	haplot. tests^a^	SL tests^b^	haplot. tests^a^	SL tests^b^	haplot. tests^a^	SL tests^b^	haplot. tests^a^	SL tests^b^
C3AR1	5	5	4	4	1	3	1	1	1	1
CCR2	5	5	1	4	1	1	1	3	1	1
CEBPB	5	4	5	4	1	1	2	2	3	3
CSF2	4	5	5	4	1	1	1	3	1	1
FCN3	5	5	1	1	1	1	1	1	1	1
FGL2	1	1	1	1	1	1	1	1	1	1
IFNG	5	5	4	3	2	1	1	4	2	1
IL13	3	5	5	4	4	1	2	3	1	1
IL24	5	5	2	1	1	4	2	1	2	1
IL9	1	2	5	4	1	2	4	5	1	1
LTA	4	5	4	4	1	1	1	3	3	2
LTB	5	5	1	1	1	1	1	1	1	1
MC1R	2	5	4	3	3	4	1	2	4	1
PLAU	4	5	5	4	3	3	1	2	2	1
PROCR	1	1	1	1	1	1	1	1	1	1
RELA	5	5	1	4	1	1	1	1	1	1
SERPINC1	4	5	5	1	1	2	2	4	3	3
TNF	1	1	4	4	3	3	2	2	4	5
TRADD	5	5	3	4	3	1	1	2	1	2
VTN	4	4	2	2	5	5	1	1	2	2

total	74	83	63	58	36	38	28	43	36	31
final rank	5	5	4	4	2	2	1	3	2	1

^a^haplotype tests

^b^Single Locus tests

**Figure 1:**
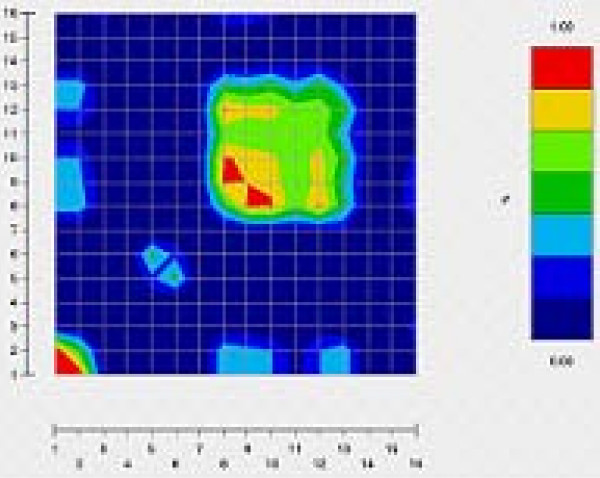
**Estimation of the LD for all the pairs of SNPs within IL13**. The LD was measured by the squared standardized coefficient r^2 ^[10]. The LD values were calculated by first estimating the frequencies of the haplotypes obtained from all the SNPs. These frequencies have then been added to infer the haplotype frequency, and further the LD value, of each pair of SNPs. All the values were color-coded with the GOLD program [11].

**Figure 2:**
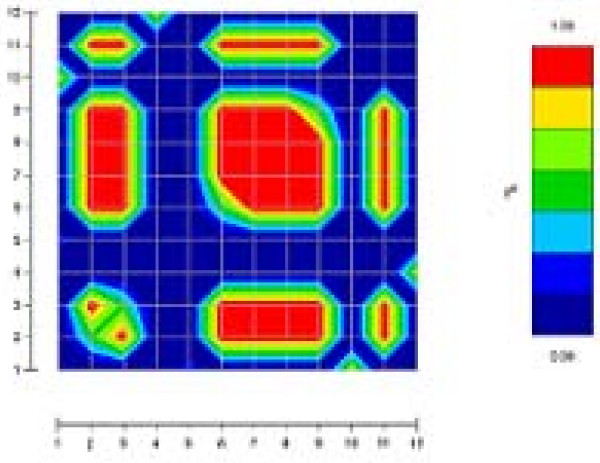
**Estimation of the LD for all the pairs of SNPs within TNF**. The LD was measured by the squared standardized coefficient r^2 ^[10]. The LD values were calculated by first estimating the frequencies of the haplotypes obtained from all the SNPs. These frequencies have then been added to infer the haplotype frequency, and further the LD value, of each pair of SNPs. All the values were color-coded with the GOLD program [11].

In order to better understand these results, we will focus on two genes that give very different results: IL13 and TNF. As shown in Figures 1 and 2, IL13 and TNF are two genes with very different patterns of LD. The amount of LD is much more important in TNF where 7 SNPs are in complete association (r^2 ^= 1) than in IL13 where no such block exists. Tables 4A and 4B show for IL13 and TNF respectively, the power to detect an association of the different subsets under genetic model A defined by *x *= 1 for the relative penetrance of heterozygous carrier of the DS allele as compared to homozygous carrier and and *s *= 0.5 for the relative penetrance of non-carrier as compared to homozygous carrier respectively. Three mean power values are presented depending on whether the assumed DS locus is any of the SNPs found in the gene (all possible DSL), any of the SNPs included in the selected subset (included DSL) or any of the SNPs excluded from the subset (excluded DSL).

**Table 4:** Power to detect an association under genetic model A (defined by *x *= 1 and *s *= 0.5 for the relative penetrances of heterozygous carrier of the DS allele and non carrier)

**A – IL13**				
Method	selected SNPs	mean power for	Model A	
			haplo. tests^d^	SL tests^e^

I (Johnson et al.)	1, 4, 9, 11, 15, 16	included DSL^a^	0.739	0.825
		excluded DSL^b^	0.597	0.504
		all possible DSL^c^	0.650	0.624
II (Stram et al)	1, 3, 4, 5, 7, 8, 10, 11, 13, 15, 16	included DSL	0.600	0.749
		excluded DSL	0.620	0.627
		all possible DSL	0.606	0.710
III (Cousin et al.)	1, 3, 4, 5, 7, 8, 11, 13, 14, 15, 16	included DSL	0.579	0.694
		excluded DSL	0.739	0.767
		all possible DSL	0.628	0.716
IV 0.5 (Carlson et al.)	1, 3, 4, 5, 6, 7, 10, 14, 15, 16	included DSL	0.526	0.612
		excluded DSL	0.905	0.914
		all possible DSL	0.668	0.725
IV 0.8 (Carlson et al.)	1, 3, 4, 5, 6, 7, 8, 13, 14, 15, 16	included DSL	0.573	0.637
		excluded DSL	0.914	0.946
		all possible DSL	0.679	0.734

**B – TNF**				

Method	selected SNPs	mean power for	Model A	
			haplo. tests^d^	SL tests^e^

I (Johnson et al.)	1, 2, 5, 12	included DSL^a^	0.909	0.930
		excluded DSL^b^	0.914	0.930
		all possible DSL^c^	0.908	0.926
II (Stram et al)	1, 4, 5, 6, 12	included DSL	0.899	0.928
		excluded DSL	0.893	0.921
		all possible DSL	0.896	0.924
III (Cousin et al.)	1, 2, 4	included DSL	0.958	0.966
		excluded DSL	0.857	0.866
		all possible DSL	0.882	0.891
IV 0.5 (Carlson et al.)	2, 4, 5, 10	included DSL	0.902	0.922
		excluded DSL	0.910	0.925
		all possible DSL	0.908	0.924
IV 0.8 (Carlson et al.)	1, 2, 4, 5, 10, 12	included DSL	0.911	0.932
		excluded DSL	0.880	0.910
		all possible DSL	0.896	0.921

^a^SNP included in the selected subset and considered as Disease Susceptibility Locus

^b^SNP excluded from the selected subset and considered as Disease Susceptibility Locus

^c^all the possible Disease Susceptibility Loci (within or outside of the subset)

^d^haplotype tests

^e^Single Locus tests

SNP subsets selected within IL13 are very different in terms of size (ranging from 6 SNPs for Method I to 11 SNPs for Methods II, III and IV with a 0.8 threshold). Contrary to what could have been expected, we did not found that the power was systematically higher when the disease susceptibility locus was included in the set of markers tested. This is due to the fact that the numbers reported are average values of the power over the different possible DS loci and for some of these loci with low DS allele frequencies (see for example SNP3 and SNP7 in IL13), power is very low. It is then difficult to compare the results over the 3 different situations (included DSL, excluded DSL and all DSL) and we thus decided to compare them within these categories. When the DSL is included in the subset of markers, we found that the subset selected by Method I is the most powerful for both types of test. As the set contains only 6 SNPs, the number of haplotypes is limited and the haplotype test more effective to detect the SNPs of the subset than the one performed with the other selected sets, in which there are at least 10 SNPs. In the same way, the correction for multiple testing is less important and the single locus tests are thus more powerful. However, in the excluded DSL category, Method I (Johnson et al.) turns out to be the one selecting the less powerful subset. In limiting the number of SNPs, the method looses a lot of information for the detection of the SNPs outside of the subset. The selections proposed by Method II (Stram et al.) and Method III (Cousin et al.) appear to be less relevant than the one proposed by Method IV (0.8 threshold) since with a same number of SNPs selected, the sets are finally less powerful to detect an association.

For TNF, selected subsets are much closer to each other in terms of size and power than for IL13. Because of the large amount of LD within the gene, the size of the subsets proposed by the different methods is limited. Method I and Method IV (with a 0.5 threshold) select the same number of SNPs. These subsets however differ in two SNPs: SNP 1 and SNP 12 for Method I and SNP 4 and SNP 10 for Method IV. If both subsets are equally powerful for the haplotype test, SNP 1 and SNP 12, which are in fact more frequent, make the subset selected by Method I more powerful for single locus tests.

## Discussion

In view of the results obtained on twenty candidate genes, it appears that none of the four selection methods we studied stands out systematically from the others. The differences between the power values obtained for a same gene with the SNP subsets selected by the different methods are indeed often very limited: on average there is a 1.8% (± 1.4) difference for haplotypic tests and a 2.8% (± 2.0) difference for single locus tests. Both methods based on haplotype information (Methods I and II) select subsets that lead to very similar power to detect an association but Method I (Johnson et al.) turns out to be much more interesting since it reaches the same levels of power with smaller subsets. Both methods based on pairwise information are also very close to each other in terms of power, with more or less efficiency in haplotypic and single locus tests according to the number of SNPs within the subsets and to the threshold used with Method IV (Carlson et al.). Method I is the best at minimizing the size of the subset but at the end, if we consider both the number of SNPs selected and the power of these markers to detect an association, Method III (Cousin et al.) turns out to be the optimal one since it ranks well for both the number of SNPs selected and the average power achieved.

Power computations are very dependent on sample sizes and we found that the ranking of the methods may substantially vary with sample sizes especially when the power obtained with the different subsets of markers were very similar. This is well illustrated in Additional File 3 for the case of TNF. Indeed, if instead of considering a sample size of 500 cases and 500 controls, we reduce the sample size by half (250 cases and 250 controls), we found that, as expected, powers are reduced but also Method III (Cousin et al.) now scores first when considering all possible DSL where it previously did not score as well.

The power computations are also sensitive to the way haplotype frequencies and pairwise LD values are estimated. In our computations, pairwise LD values were obtained after estimation of haplotype frequencies on all SNPs and summation over haplotypes carrying the same alleles at the two studied loci. If instead we had estimated haplotypes using only the two-locus information, the power values obtained could have been more or less different depending on the genetic model considered. However, because the impact is the same on the four methods, the ranking of the methods remains unchanged.

In this study, we always assumed for the computations that susceptibility was due to a single DS locus. It would be interesting in a future study to consider genetic models with several DS loci within the same candidate gene.

For all the selections, both methods based on haplotype information were applied on all the haplotypes. As often suggested with these methods, we could also have only considered common haplotypes. This would have lead to the selection of smaller subsets but also to a loss of power when considering DS loci with low allele frequencies, as shown recently by Zhang et al. [12]. Nevertheless, it would have not changed our conclusions since Method I (Johnson et al.) is already the one selecting the smaller number of SNPs and already, as well as Method II (Stram et al.), the one selecting the less powerful subsets.

Our comparative study is not exhaustive but most of the selection methods recently proposed in the literature [13-15] are based either on haplotype information or on the definition of LD groups. The methods we included in our study appeared to us as the most representative. Finally, other criteria, such as the genomic properties of the SNPs could also be considered for SNP selection. Some new methods rely mostly on this type of criteria [16,17]. This is also already integrated in Method IV (Carlson et al.), since if information on the genomic nature of the SNPs (coding, non coding ...) is available, we can use it to choose among the different subsets proposed by the method. It could be integrated in the selection with Method III by weighting the probability for the different loci within the gene to be DS loci. However, as this type of criteria is not considered for selection with the methods based on haplotype information, we did not use it for our comparative study.

## Conclusion

In conclusion, as shown with the different results obtained on twenty candidate genes, the choice of the optimal selection method is not obvious. Both methods based on pairwise LD information, and especially Method III (Cousin et al.), turn out to be the most interesting methods in this context of association study in candidate gene. In a context where the number of SNPs to be tested in a given region needs to be more limited, as in large-scale studies or wide genome scans, Method I (Johnson et al.) would probably be more suitable as it selects significantly less markers than the other methods.

## Methods

### Methods based on haplotype information

Method I, the haplotype tag SNP (htSNP) method proposed by Johnson et al. [5], identifies the set of markers that best captures the haplotype information. The selection is based on statistics related to diversity criteria: the proportion of haplotype diversity explained by the htSNPs and the residual diversity, measuring how well these htSNPs can predict the markers excluded from the set. For a same number of htSNPs, the best subset is the one that best maximises the overall percentage of haplotype diversity observed while minimising the residual diversity. The number of htSNPs to keep is then determined by comparing the diversity values of the best subset of each size. The smallest subset that scores well the different statistics will be the one finally chosen. The thresholds we fixed for the htSNP subset selection were a minimum of 85% of diversity explained and a maximum of 0.05 of mean residual diversity.

As Method I, the tagSNP method developed by Stram et al. [6] and referred to as Method II, aims at identifying SNPs that best represent the haplotype structure of the gene or region. It selects the SNPs that optimize the predictability of the haplotypes. The selection is based on the calculation of a statistics, R_*h*_^2^, measuring the correlation between the true frequency of haplotype *h *and the one that could be predicted from a subset of markers. For a given subset size, the best set of markers is defined as the one that maximizes the minimum R_*h*_^2^. As expected, values of R_*h*_^2 ^increase with the number of markers included in the subset. The number of tagSNPs to select is finally decided by fixing a threshold for R_*h*_^2 ^(here we chose a threshold of 90%) and identifying the smallest subset with a minimum R_*h*_^2 ^exceeding this threshold.

For both methods, we used a minimum haplotype frequency cut-off of 0; i.e. we did not discard rare haplotypes.

### Methods based on pairwise information

The selection method proposed by Cousin et al. [7] and referred to as Method III, is based on the pairwise linkage disequilibrium between the different SNPs of the gene and on their allele frequencies. To select the best set of SNPs, power computations are performed for a wide range of penetrance values assuming that each of the *M *typed polymorphisms within the gene can equally be the DS locus. A Bonferroni correction is applied for multiple testing and an average power is estimated over all DS loci and penetrance values. For a given number of selected markers, the best subset is then defined as the one with the best average power. Power is expected to increase with increasing numbers of markers in the subsets. However, because of the Bonferroni correction, power increases up to a maximum value, reached for a given size *n*, and then decreases. Method III therefore considers that the optimal number of SNPs to select is *n*.

In Method IV, developed by Carlson et al. [8], the selection also relies on linkage disequilibrium and more specifically on r^2^, the squared standardized coefficient [11]. At first, bins of SNPs are defined by grouping together SNPs with r^2 ^values that exceed a chosen threshold. All the SNPs within a same bin are not necessarily in strong LD since if SNP A exceeds the r^2 ^threshold with SNP B and SNP C, this might be untrue for the pair SNP B/SNP C. The markers exceeding the r^2 ^threshold with all the markers of the bin are the ones designated as tagSNP. Several tagSNPs may be designated within a same bin and in a second step, the user can then refine the selection using different criteria such as the genomic properties of the markers. Two different r^2 ^thresholds were considered in our study: 0.5 and 0.8.

### The genotype data

The four selection methods were applied to the genotype data on twenty candidate genes sequenced on 23 European Americans (data available at the University of Washington-Fred Hutchinson Cancer Research Center Web site [9]). These genes were chosen in order to have different number of polymorphisms and different linkage disequilibrium patterns. As shown in Table 1 and Additional file 1, the twenty candidate genes had between 6 and 27 SNPs with various allele frequencies. Patterns of LD were also very different from one gene to another: some had very high level of LD among their SNPs (like TNF for example, with 33% of the SNP pairs in strong LD, i.e. r^2 ^> 0.70), whereas genes like IL13 had very low levels of LD.

Allele and haplotype frequencies obtained on these data were used in the power computations as the frequencies in controls.

### Power computations

We based the comparison of the four selection methods on the power to detect an association that would be expected under different genetic models with the different sets of selected markers.

The power of a given subset under a specific genetic model was determined by estimating the asymptotic power of the homogeneity chi-square of both the haplotypic test and the single locus test. Allele and haplotype frequencies in controls were calculated from the genotype data retrieved on the University of Washington-Fred Hutchinson Cancer Research Center Web site [9] using the EM algorithm SNPHAP [18]. Expected haplotype and allele frequencies in cases were then derived under the assumption of a single disease susceptibility locus. Let locus *i *be the disease susceptibility locus and allele *a *at this locus of frequency *p*_*a *_be the one conferring an increase risk of disease. *Let G*_*a *_be the set of haplotypes carrying this allele *a *and *G_A_*, the set of haplotypes carrying the other allele *A *at locus *i*. Let *p [h_*1*_]*_(*Ga*) _and *p [h_*1*_]*_(*GA*) _be respectively the frequency of haplotype *h*_*1 *_in *G*_*a *_and *G*_*A *_and p [h_1_*]_(*Ga*) _and p[h_1_*]_(*GA*)_, the sum of the frequencies of all the other haplotypes in *G*_*a *_and *G*_*A*_. The expected frequency of haplotype *h*_*1 *_in cases is then:

P[h1/affected]=(1)+(2)+(3)K
 MathType@MTEF@5@5@+=feaafiart1ev1aaatCvAUfKttLearuWrP9MDH5MBPbIqV92AaeXatLxBI9gBaebbnrfifHhDYfgasaacH8akY=wiFfYdH8Gipec8Eeeu0xXdbba9frFj0=OqFfea0dXdd9vqai=hGuQ8kuc9pgc9s8qqaq=dirpe0xb9q8qiLsFr0=vr0=vr0dc8meaabaqaciaacaGaaeqabaqabeGadaaakeaacqWGqbaudaWadaqaaiabdIgaOnaaBaaaleaacqaIXaqmaeqaaOGaei4la8IaemyyaeMaemOzayMaemOzayMaemyzauMaem4yamMaemiDaqNaemyzauMaemizaqgacaGLBbGaayzxaaGaeyypa0ZaaSaaaeaadaqadaqaaiabigdaXaGaayjkaiaawMcaaiabgUcaRmaabmaabaGaeGOmaidacaGLOaGaayzkaaGaey4kaSYaaeWaaeaacqaIZaWmaiaawIcacaGLPaaaaeaacqWGlbWsaaaaaa@4942@

with

(1)=(p[h1](Ga)p[h1*](Ga)+p[h1](Ga)p[h1](Ga))(2)=(p[h1](Ga)p[h1*](GA)+p[h1](GA)p[h1*](Ga)+2p[h1](GA)p[h1](Ga))x(3)=(p[h1](GA)p[h1](GA)+p[h1](GA)p[h1*](GA))sK=pa2+2pa(1−pa)x+(1−pa)2s
 MathType@MTEF@5@5@+=feaafiart1ev1aaatCvAUfKttLearuWrP9MDH5MBPbIqV92AaeXatLxBI9gBaebbnrfifHhDYfgasaacH8akY=wiFfYdH8Gipec8Eeeu0xXdbba9frFj0=OqFfea0dXdd9vqai=hGuQ8kuc9pgc9s8qqaq=dirpe0xb9q8qiLsFr0=vr0=vr0dc8meaabaqaciaacaGaaeqabaqabeGadaaakeaafaqabeabbaaaaeaadaqadaqaaiabigdaXaGaayjkaiaawMcaaiabg2da9maabmaabaGaemiCaa3aamWaaeaacqWGObaAdaWgaaWcbaGaeGymaedabeaaaOGaay5waiaaw2faamaaBaaaleaacqGGOaakcqWGhbWrcqWGHbqycqGGPaqkaeqaaOGaemiCaaNaei4waSLaemiAaG2aa0baaSqaaiabigdaXaqaaiabcQcaQaaakiabc2faDnaaBaaaleaacqGGOaakcqWGhbWrcqWGHbqycqGGPaqkaeqaaOGaey4kaSIaemiCaa3aamWaaeaacqWGObaAdaWgaaWcbaGaeGymaedabeaaaOGaay5waiaaw2faamaaBaaaleaacqGGOaakcqWGhbWrcqWGHbqycqGGPaqkaeqaaOGaemiCaa3aamWaaeaacqWGObaAdaWgaaWcbaGaeGymaedabeaaaOGaay5waiaaw2faamaaBaaaleaacqGGOaakcqWGhbWrcqWGHbqycqGGPaqkaeqaaaGccaGLOaGaayzkaaaabaWaaeWaaeaacqaIYaGmaiaawIcacaGLPaaacqGH9aqpdaqadaqaaiabdchaWnaadmaabaGaemiAaG2aaSbaaSqaaiabigdaXaqabaaakiaawUfacaGLDbaadaWgaaWcbaGaeiikaGIaem4raCKaemyyaeMaeiykaKcabeaakiabdchaWjabcUfaBjabdIgaOnaaDaaaleaacqaIXaqmaeaacqGGQaGkaaGccqGGDbqxdaWgaaWcbaGaeiikaGIaem4raCKaemyqaeKaeiykaKcabeaakiabgUcaRiabdchaWnaadmaabaGaemiAaG2aaSbaaSqaaiabigdaXaqabaaakiaawUfacaGLDbaadaWgaaWcbaGaeiikaGIaem4raCKaemyqaeKaeiykaKcabeaakiabdchaWjabcUfaBjabdIgaOnaaDaaaleaacqaIXaqmaeaacqGGQaGkaaGccqGGDbqxdaWgaaWcbaGaeiikaGIaem4raCKaemyyaeMaeiykaKcabeaakiabgUcaRiabikdaYiabdchaWnaadmaabaGaemiAaG2aaSbaaSqaaiabigdaXaqabaaakiaawUfacaGLDbaadaWgaaWcbaGaeiikaGIaem4raCKaemyqaeKaeiykaKcabeaakiabdchaWnaadmaabaGaemiAaG2aaSbaaSqaaiabigdaXaqabaaakiaawUfacaGLDbaadaWgaaWcbaGaeiikaGIaem4raCKaemyyaeMaeiykaKcabeaaaOGaayjkaiaawMcaaiabdIha4bqaamaabmaabaGaeG4mamdacaGLOaGaayzkaaGaeyypa0ZaaeWaaeaacqWGWbaCdaWadaqaaiabdIgaOnaaBaaaleaacqaIXaqmaeqaaaGccaGLBbGaayzxaaWaaSbaaSqaaiabcIcaOiabdEeahjabdgeabjabcMcaPaqabaGccqWGWbaCdaWadaqaaiabdIgaOnaaBaaaleaacqaIXaqmaeqaaaGccaGLBbGaayzxaaWaaSbaaSqaaiabcIcaOiabdEeahjabdgeabjabcMcaPaqabaGccqGHRaWkcqWGWbaCdaWadaqaaiabdIgaOnaaBaaaleaacqaIXaqmaeqaaaGccaGLBbGaayzxaaWaaSbaaSqaaiabcIcaOiabdEeahjabdgeabjabcMcaPaqabaGccqWGWbaCcqGGBbWwcqWGObaAdaqhaaWcbaGaeGymaedabaGaeiOkaOcaaOGaeiyxa01aaSbaaSqaaiabcIcaOiabdEeahjabdgeabjabcMcaPaqabaaakiaawIcacaGLPaaacqWGZbWCaeaacqWGlbWscqGH9aqpcqWGWbaCdaqhaaWcbaGaemyyaegabaGaeGOmaidaaOGaey4kaSIaeGOmaiJaemiCaa3aaSbaaSqaaiabdggaHbqabaGcdaqadaqaaiabigdaXiabgkHiTiabdchaWnaaBaaaleaacqWGHbqyaeqaaaGccaGLOaGaayzkaaGaemiEaGNaey4kaSYaaeWaaeaacqaIXaqmcqGHsislcqWGWbaCdaWgaaWcbaGaemyyaegabeaaaOGaayjkaiaawMcaamaaCaaaleqabaGaeGOmaidaaOGaem4Camhaaaaa@F174@

where *x *is the relative penetrance of heterozygous carrier of the DS allele as compared to homozygous carrier and *s*, the relative penetrance of non-carrier as compared to homozygous carrier.

In the same way, we estimated for the single locus tests, the expected frequency of the marker allele *m *in cases:

P(m|affected)=pmCampa+[Cam(1−pm)+(1−Cam)pm]x+(1−Cam)(1−pa)spa2+2pa(1−pa)x+(1−pa)2s
 MathType@MTEF@5@5@+=feaafiart1ev1aaatCvAUfKttLearuWrP9MDH5MBPbIqV92AaeXatLxBI9gBaebbnrfifHhDYfgasaacH8akY=wiFfYdH8Gipec8Eeeu0xXdbba9frFj0=OqFfea0dXdd9vqai=hGuQ8kuc9pgc9s8qqaq=dirpe0xb9q8qiLsFr0=vr0=vr0dc8meaabaqaciaacaGaaeqabaqabeGadaaakeaacqWGqbaudaqadaqaaiabd2gaTjabcYha8jabdggaHjabdAgaMjabdAgaMjabdwgaLjabdogaJjabdsha0jabdwgaLjabdsgaKbGaayjkaiaawMcaaiabg2da9iabdchaWnaaBaaaleaacqWGTbqBaeqaaOWaaSaaaeaacqWGdbWqdaWgaaWcbaGaemyyaeMaemyBa0gabeaakiabdchaWnaaBaaaleaacqWGHbqyaeqaaOGaey4kaSYaamWaaeaacqWGdbWqdaWgaaWcbaGaemyyaeMaemyBa0gabeaakmaabmaabaGaeGymaeJaeyOeI0IaemiCaa3aaSbaaSqaaiabd2gaTbqabaaakiaawIcacaGLPaaacqGHRaWkdaqadaqaaiabigdaXiabgkHiTiabdoeadnaaBaaaleaacqWGHbqycqWGTbqBaeqaaaGccaGLOaGaayzkaaGaemiCaa3aaSbaaSqaaiabd2gaTbqabaaakiaawUfacaGLDbaacqWG4baEcqGHRaWkdaqadaqaaiabigdaXiabgkHiTiabdoeadnaaBaaaleaacqWGHbqycqWGTbqBaeqaaaGccaGLOaGaayzkaaWaaeWaaeaacqaIXaqmcqGHsislcqWGWbaCdaWgaaWcbaGaemyyaegabeaaaOGaayjkaiaawMcaaiabdohaZbqaaiabdchaWnaaDaaaleaacqWGHbqyaeaacqaIYaGmaaGccqGHRaWkcqaIYaGmcqWGWbaCdaWgaaWcbaGaemyyaegabeaakmaabmaabaGaeGymaeJaeyOeI0IaemiCaa3aaSbaaSqaaiabdggaHbqabaaakiaawIcacaGLPaaacqWG4baEcqGHRaWkdaqadaqaaiabigdaXiabgkHiTiabdchaWnaaBaaaleaacqWGHbqyaeqaaaGccaGLOaGaayzkaaWaaWbaaSqabeaacqaIYaGmaaGccqWGZbWCaaaaaa@8BEF@

where *C*_*am *_is the probability to observe allele *a *at the DS locus given allele *m *at the marker.

A sample size of 500 cases and 500 controls was considered for the calculations. We studied a range of 55 genetic models obtained by considering all possible penetrance values *x *and *s *between 0 and 1 (by increment of 0.1 and imposing that *s *≤ *x*). We also assumed that each of the *n *SNPs of the gene had an equal chance of being the susceptibility locus. For all the DS loci, we assumed that the DS allele was the minor allele. For both tests, the power of detection of an association was then for a given subset, the average power value estimated over all DS loci and penetrance values.

Power computations for single locus tests were then based on the same principle as the ones used for SNP selection in Method III (Cousin et al.). Unlike this method, which uses Bonferroni correction for multiple testing, single locus tests performed here to measure the "efficiency" of the four methods were corrected for multiple testing by the SNPSpD program developed by Nyholt [19].

## Authors' contributions

EC performed the comparative study, contributed to the conception of the study and wrote the manuscript. JFD contributed to the design of the study. EG contributed to the design and the conception of the study and to the manuscript preparation. All authors read and approved the final manuscript.

## Supplementary Material

Additional File 1 Main characteristics of the twenty candidate genes Click here for file

Additional File 2 Power of the selected subsets to detect an association Click here for file

Additional File 3 Power results for TNF under Model A and with a sample size of 250 cases and 250 controls Click here for file
