# Supplementary material for: Selection of SNP subsets for association studies in candidate genes: comparison of the power of different strategies to detect single disease susceptibility locus effects
Source: BMC Genet. 2006 Apr 5;7:20. doi: 10.1186/1471-2156-7-20 (PMC1458358; doi:10.1186/1471-2156-7-20)
Supplement: Additional File 1 — Main characteristics of the twenty candidate genes [file 1471-2156-7-20-S1.doc]

## Main characteristics of the twenty candidate genes
